# Balancing Energy Consumption and Detection Accuracy in Cardiovascular Disease Diagnosis: A Spiking Neural Network-Based Approach with ECG and PCG Signals

**DOI:** 10.3390/s25175263

**Published:** 2025-08-24

**Authors:** Guihao Ran, Yijing Wang, Han Zhang, Jiahui Cheng, Dakun Lai

**Affiliations:** School of Electronic Science and Engineering, University of Electronic Science and Technology of China, Chengdu 611731, China

**Keywords:** electrocardiogram, phonocardiogram, cardiovascular diseases, spiking neural networks, energy consumption

## Abstract

Electrocardiogram (ECG) and phonocardiogram (PCG) signals are widely used in the early prevention and diagnosis of cardiovascular diseases (CVDs) due to their ability to accurately reflect cardiac conditions from different physiological perspectives and their ease of acquisition. Currently, some studies have explored the joint use of ECG and PCG signals for disease screening, but few studies have considered the trade-off between classification performance and energy consumption in model design. In this study, we propose a multimodal CVDs detection framework based on Spiking Neural Networks (SNNs), which integrates ECG and PCG signals. A differential fusion strategy at the signal level is employed to generate a fused EPCG signal, from which time–frequency features are extracted using the Adaptive Superlets Transform (ASLT). Two separate Spiking Convolutional Neural Network (SCNN) models are then trained on the ECG and EPCG signals, respectively. A confidence-based dynamic decision-level (CDD) fusion strategy is subsequently employed to perform the final classification. The proposed method is validated on the PhysioNet/CinC Challenge 2016 dataset, achieving an accuracy of 89.74%, an AUC of 89.08%, and an energy consumption of 209.6 μJ. This method not only achieves better balancing performance compared to unimodal signals but also realizes an effective balance between model energy consumption and classification effect, which provides an effective idea for the development of low-power, multimodal medical diagnostic systems.

## 1. Introduction

Cardiovascular diseases (CVDs) are the leading cause of death globally, accounting for approximately 32% of all deaths worldwide. Their high prevalence, disability rate, and associated medical burden pose a significant challenge to public health systems [1,2]. Consequently, there has been growing emphasis in recent years on the early detection and diagnosis of CVDs. Among various diagnostic methods, physiological signals play a critical role, particularly the electrocardiogram (ECG) [3,4,5,6,7] and the phonocardiogram (PCG) [8,9,10]. These signals can accurately reflect the state of the heart from multiple perspectives and are easily acquired through non-invasive and convenient means, making them well-suited for large-scale population screening and long-term monitoring [11].

Most early detection studies of CVDs rely on unimodal signal analysis, focusing either on ECG or PCG data. These approaches can be broadly categorized into traditional feature engineering combined with machine learning and end-to-end deep learning methods. For example, Zhu et al. [12] extracted morphological features (e.g., amplitude and duration) from the P-QRS-T waveforms of ECG signals, then applied Principal Component Analysis (PCA) for dimensionality reduction and Dynamic Time Warping (DTW) for temporal feature extraction, feeding the results into a Support Vector Machine (SVM) classifier. Kui et al. [13], focusing on PCG signals, employed an improved Duration-Dependent Hidden Markov Model (DD-HMM) to segment cardiac cycles and extracted Mel-Frequency Spectral Coefficient (MFSC) features, which were subsequently classified using a Convolutional Neural Network (CNN). In contrast, deep learning methods have shifted toward end-to-end architectures. For instance, Kusuma et al. [14] proposed a hybrid Deep Convolutional Neural Network–Long Short-Term Memory (DCNN-LSTM) model, where CNNs are used to extract spatial features from ECG signals and LSTMs are applied to model temporal dynamics, enabling the diagnosis of Congestive Heart Failure (CHF). Alkhodari et al. [15] employed a Convolutional Neural Network–Bidirectional Long Short-Term Memory (CNN-BiLSTM) model to detect cardiovascular abnormalities and diagnose valvular diseases from PCG signals, leveraging the bidirectional LSTM and its enhanced context modeling capabilities.

Although the aforementioned unimodal approaches based on either ECG or PCG signals have achieved notable progress in CVDs detection, they are inherently limited by the physiological differences in signal origin—PCG reflects mechanical cardiac activity, while ECG captures electrical activity. As a result, single-modality analysis is often insufficient to comprehensively evaluate cardiac function. In response, multimodal fusion has emerged as a promising trend in recent research. For example, Li et al. [16] extracted features from both ECG and PCG signals using a traditional artificial neural network, applied a genetic algorithm to select optimal feature subsets, and employed an SVM classifier for final prediction. Zhu et al. [17] proposed DDR-Net, a dual-domain representation network that integrates multiscale low-level features from ECG and PCG signals using a dual-scale feature aggregation module. This is followed by SVM-RFECV-based feature selection and classification using an SVM, enabling efficient and accurate CVDs detection. These studies demonstrate that multimodal fusion strategies significantly enhance the comprehensiveness and robustness of CVDs detection systems, offering a more holistic representation of cardiac function.

However, most existing multimodal signal-based studies rely on conventional artificial neural networks (ANNs), which present several limitations. Specifically, when the network architecture is simple and thus consumes less energy, the model performance is often suboptimal. Conversely, more complex ANN models can improve performance but at the cost of significantly increased energy consumption. Furthermore, with the ongoing expansion of data volumes and network scales, ANN-based models demand substantial computational resources and power, resulting in slower inference speeds. This poses a particular challenge for wearable and edge devices, where efficient real-time deployment is critical [18]. Therefore, it is imperative to explore methods that can effectively balance limited computational resources with acceptable model performance.

Based on the above analysis, this study proposes an innovative CVDs detection framework that leverages Spiking Neural Networks (SNNs) and incorporates both signal-level and decision-level fusion of ECG and PCG multimodal signals. From a network perspective, SNNs offer significantly lower energy consumption than conventional ANNs due to their inherent sparsity and event-driven processing characteristics [19,20,21]. In terms of fusion strategy, this work adopts a hybrid approach that combines signal-level and decision-level fusion, which ensures a balance between detection performance and computational efficiency. Compared to conventional feature-level fusion, this approach substantially reduces computational overhead while maintaining classification accuracy. It is worth noting that although there has been preliminary exploration of SNN in CVDs detection—for example, Rana et al. [22] proposed the use of SNN with attention mechanisms to enhance feature extraction from ECG signals, effectively leveraging the efficiency of SNN and the precision of attention modules to improve cardiac signal analysis—existing studies remain largely focused on unimodal signal analysis. There is still a pressing need to further investigate the application of SNN-based multimodal signal fusion for more effective and efficient CVDs detection.

Specifically, this study first performs signal-level fusion of ECG and PCG signals, followed by time–frequency transformation using the Adaptive Superlets Transform (ASLT) to obtain high-resolution spectrograms. The transformed signals are then separately fed into SNN models for training. Finally, a decision-level fusion of the trained models is carried out to classify and detect CVDs. This framework successfully explores an effective balance between model energy consumption and classification performance, providing valuable insights for the development of low-power, multimodal medical diagnostic systems.

## 2. Materials and Methods

### 2.1. Framework

**Figure 1:**
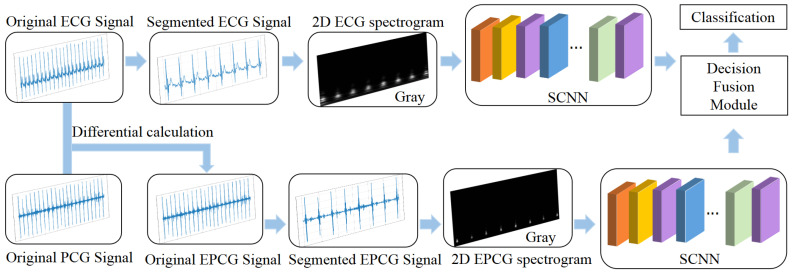
The framework of multimodal model for predicting CVDs.

Figure 1 illustrates the architecture of the proposed model, which predicts CVDs by integrating original ECG signals and signal-level fused ECG-PCG signals (referred to as EPCG). The framework consists of four main stages: signal segmentation, time–frequency transformation, network training, and final decision-level fusion for classification. Specifically, after data segmentation and preprocessing, the ASLT is applied to convert the time-series signals into time–frequency spectrograms. These spectrograms, derived from the ECG and EPCG signals in the training set, are then used to independently train two Spiking Convolutional Neural Networks (SCNNs) using five-fold cross-validation. After training, the corresponding ECG and EPCG spectrograms from the test set are input into the two trained models for inference. Finally, a decision-level fusion module combines the outputs of the two models to produce the final classification result.

### 2.2. Datasets

The dataset used in this study is derived from the PhysioNet/CinC Challenge 2016 [23], contributed by multiple international institutions. Based on the source institutions, the dataset is divided into six subsets, labeled from “training-a” to “training-f”. Among them, the “training-a” subset contains simultaneous ECG and PCG recordings collected from the same subjects. Both ECG and PCG signals are sampled at 2000 Hz.

Specifically, the “training-a” subset includes a total of 409 recordings, of which 405 contain both ECG and PCG signals. Within these 405 recordings, 117 are labeled as negative (normal controls), while the remaining 288 are labeled as positive, corresponding to patients diagnosed with mitral valve prolapse (MVP), benign aortic disease (AD), or other miscellaneous pathological conditions (MPCs). The duration of the recordings ranges from 9.27 s to 36.5 s. Due to severe noise contamination in some signals, making their classification unreliable, 17 recordings were excluded from further analysis [24], leaving 388 usable recordings.

To enable signal-level fusion of ECG and PCG, it was necessary to ensure that both signals were of equal length. Therefore, we selected 382 recordings from the remaining 388 that contained more than 40,000 data points, ensuring sufficient signal duration without significantly reducing the dataset size. For each selected recording, we extracted a 36,000-point segment from the 4000th to the 40,000th sample, to avoid unstable signal portions typically occurring at the beginning of data acquisition.

Finally, due to the limited number of samples and class imbalance, we applied a data augmentation strategy [25]. Specifically, each signal was segmented into 6-second segments using a sliding window approach. A step size of 2 s was used for normal samples and 6 s for abnormal samples. The 6-second duration was chosen to ensure the inclusion of multiple complete cardiac cycles while allowing accurate segmentation of the entire dataset and generating a sufficient number of samples for model training. This process resulted in a total of 1554 samples as summarized in Table 1.

### 2.3. Adaptive Superlets Transform (ASLT)

Adaptive Superlets Transform (ASLT) [26] enhances the time–frequency resolution by dynamically adjusting the number of wavelets, achieving an adaptive trade-off between time and frequency localization. Compared with traditional fixed-wavelet methods, ASLT is based on the concept of adaptive super-resolution. The Adaptive Superlet (ASL) combines a series of small wavelets centered around a given frequency, where the number of wavelets is adjusted depending on the frequency. Its general form is given by

(1) $$ASL_{f}=SL_{f,o}|o-a\left(f\right)$$

where a(f) is a function related to the frequency f, used to determine the number of small wavelets at different frequencies. In practical applications, this function is typically defined as

(2) $$a\left(f\right)=o_{\min}+\left(o_{\max}-o_{\min}\right)\cdot \frac{f-f_{\min}}{f_{\max}-f_{\min}}$$

where $o_{\min}$ and $o_{\max}$ represent the orders corresponding to the lowest and highest center frequencies, respectively, while $f_{\min}$ and $f_{\max}$ define the lower and upper bounds of the frequency range under analysis.

To address the "banding effect" caused by discrete jumps in wavelet order, fractional superlets introduce a weighted geometric mean, allowing the order to vary continuously. The response is computed as follows:

(3) $$R\left[SL_{f,o}\right]=\sqrt[{o}]{\prod_{i=1}^{o}R\left[\psi_{f,c_{i}}\right]}$$

where $R\left[\psi_{f,c_{i}}\right]$ denotes the response of a single wavelet, which is computed via complex convolution:

(4) $$R\left[\psi_{f,c_{i}}\right]=\sqrt{2}\cdot x*\psi_{f,c_{i}}$$

This enhancement enables the Fractional Adaptive Superlet Transform (FASLT) to provide a smooth representation across the entire frequency domain.

A key advantage of ASLT lies in its ability to maintain a constant absolute bandwidth configuration. By dynamically coupling the wavelet order with frequency, ASLT achieves consistent resolution performance in wideband analysis. Compared with traditional methods such as the Short-Time Fourier Transform (STFT) and Continuous Wavelet Transform (CWT), ASLT demonstrates superior performance—particularly in analyzing complex time–frequency data.

### 2.4. Spiking Convolutional Neural Network (SCNN)

**Figure 2:**
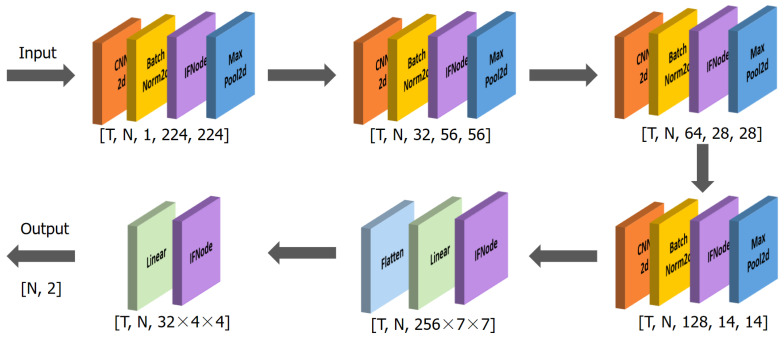
The framework of the multimodal model for predicting CVDs.

The network architecture used in this study is illustrated in Figure 2. It is a spatiotemporal feature extraction model based on SNN, with the core innovation of converting the continuous-valued operations in conventional ANN into discrete spike-based computations. This event-driven paradigm enables highly efficient inference.

Specifically, the model consists of four sequential Convolutional Spiking Blocks, followed by two fully connected spiking layers that form the classifier. The input data are expanded along the temporal dimension (repeated T times) to form spike sequences, which are then aggregated using Temporal Mean Pooling to produce the final classification output.

The spiking units in the network are implemented using Integrate-and-Fire (IF) neurons, and the spike generation process is approximated using the arctangent function (ATan) as a surrogate function [27]. This combination maintains biological plausibility while facilitating effective gradient backpropagation. The subthreshold neural dynamics of the Integrate-and-Fire Node (IFNode) are defined by the following equation:

(5) $$\frac{dV(t)}{dt}=V\left(t\right)+X\left(t\right)$$

From the perspective of discrete modeling, the subthreshold membrane dynamics of the IF neuron can be expressed as

(6) V[t]−V[t−1]=X[t]

where V[t] denotes the membrane potential of the neuron at time step *t*, and V[t−1] represents the membrane potential at the previous time step t−1. X[t] corresponds to the external input to the neuron at time step *t*.

The ATan function can be defined as follows:

(7) $$g\left(x\right)=\frac{1}{\pi }\arctan\left(\frac{\pi }{2}\alpha x\right)+\frac{1}{2}$$

where *x* denotes the input to the neuronal membrane potential or the spike function, and α is a tunable scaling factor employed to regulate the slope or smoothness of the surrogate function.

In the four convolutional blocks, the first convolutional module consists of a 7 × 7 two-dimensional convolutional layer, which expands the number of channels from 1 to 32 and employs a stride of 2 for initial spatial downsampling. The subsequent three convolutional layers progressively increase the channel dimensions to 2×, 4×, and 8× of the original size, respectively. Each of these layers utilizes a 3 × 3 convolutional kernel with a padding of one pixel to maintain the spatial resolution of the feature maps. Each convolutional layer is followed by a batch normalization layer, an IFNode, and a max-pooling layer, which respectively serve to stabilize the training process, simulate the nonlinear spiking behavior of biological neurons, and compress spatial dimensions while suppressing local noise.

After the convolutional stage, the output feature maps are flattened into a one-dimensional vector via a flatten operation and then fed into fully connected layers for further processing. The first fully connected layer maps the feature dimensions from channels × 8 × 7 × 7 to channels × 4 × 4, followed by IFNode activation. The second fully connected layer produces a 2-dimensional output representing the final classification decision, which is also paired with an IFNode to maintain the temporal response structure of the model.

### 2.5. Fusion Method

#### 2.5.1. Signal-Level Fusion

This study proposes a signal-level differential fusion method for multimodal physiological signals, grounded in the concept of differencing, as an effective approach to multi-source information integration. Specifically, a fused signal termed EPCG is constructed by performing point-wise differencing between synchronously acquired ECG and PCG signals. Due to the strong temporal synchronization between ECG and PCG signals, differential operation can effectively suppress the common background noise present in both signals. This approach is primarily based on the common-mode noise suppression principle from classical signal processing theory. Differential circuits or differential operations are important methods for improving the common-mode rejection ratio (CMRR). Furthermore, the differential operation involves only subtraction, making it a low-complexity operation. Therefore, considering both computational load and noise suppression, we propose a differential-based signal fusion method that can reduce the background noise in the fused EPCG signal.

It is worth noting that, although PCG signals hold significant clinical value for cardiac disease detection, their raw forms are generally more susceptible to noise interference and inter-individual variability, which limits their diagnostic performance in real-world applications. In contrast, ECG signals demonstrate greater stability and robustness in both temporal and morphological characteristics. Accordingly, compared to the ECG signal, the differential processing may introduce more unstable information from the PCG, potentially reducing the overall performance of the fused result. However, compared to the PCG signal, the differential processing not only suppresses certain non-stationary noise components in the PCG but also effectively integrates temporal features from the ECG. As a result, the constructed EPCG signal demonstrates higher sensitivity and discriminative capability in pathological recognition tasks than the original PCG.

This method provides a novel and effective multimodal signal fusion mechanism without significantly increasing computational complexity. It lays the foundation for subsequent decision-level fusion, preventing large discrepancies between signals from adversely affecting the final fusion outcome.

#### 2.5.2. Decision-Level Fusion

In terms of decision-level fusion, this study designs and implements a confidence-based dynamic decision (CDD) fusion strategy to integrate the classification outputs from two signal-specific models. Specifically, the strategy prioritizes the ECG-based model (Model 1), which demonstrates greater stability and robustness, and determines whether to directly adopt its prediction or incorporate the EPCG-based model (Model 2) for corrective fusion based on prediction confidence.

The strategy begins by calculating the prediction confidence of Model 1 for each sample, defined as the maximum probability from its softmax output. If this confidence exceeds a predefined threshold, the prediction from Model 1 is accepted directly, which enhances fusion efficiency and avoids unnecessary computation. When Model 1 shows insufficient confidence in its prediction, Model 2 is introduced to assist. In such cases, a normalized weighted average of the predicted probabilities from both models is computed to form a fused result, thereby improving classification robustness for ambiguous or challenging samples. The final predicted class is determined by the category with the highest value in the weighted probability distribution.

This approach realizes the dynamic scheduling of “complementary advantages” between models in terms of strategy, and the structure is simple and can improve the robustness of the overall system.

## 3. Experiment

### 3.1. Experimental Setup

The experimental dataset was partitioned into training and testing subsets in a 9:1 ratio [17]. To ensure data independence and eliminate the risk of sample leakage at its source, all data augmentation procedures were conducted independently within the training and testing sets after the split.

The model training configuration was as follows: the time step (T) was set to 6 to balance model performance and energy consumption. As shown in Table 2 , when T = 6, both the Average Validation Loss and Overall Spike Rate for ECG and EPCG signals achieve a certain balance. The Adam optimizer was employed, with an initial learning rate of 0.01. A cosine annealing learning rate schedule was adopted to dynamically adjust the learning rate, aiming to enhance convergence stability and performance. To accommodate the characteristics of different signal modalities, the number of training epochs was set differentially: 150 epochs for ECG signals and 160 epochs for EPCG signals [16]. During the decision-level fusion stage, high-reliability prediction results are screened by presetting the CDD threshold to 0.5 to effectively integrate multimodal information.

The SNN framework used in this study was SpikingJelly [27], an open-source, PyTorch-based deep learning framework. SpikingJelly provides a full-stack solution for spiking deep learning, encompassing neuromorphic data processing, deep SNN construction, surrogate gradient-based training, ANN-to-SNN conversion, weight quantization, and deployment on neuromorphic hardware.

### 3.2. Evaluation Metrics

To comprehensively evaluate the classification performance of the proposed model, five widely used metrics were adopted: Accuracy (Acc), Sensitivity (Sen), Specificity (Spe), F1-score, and Area Under the Receiver Operating Characteristic Curve (AUC). The definitions of these metrics are as follows:

(8) $$\mathrm{Acc}=\frac{TP+TN}{TP+TN+FP+FN}$$

(9) $$\mathrm{Sen}=\frac{TP}{TP+FN}$$

(10) $$\mathrm{Spe}=\frac{TN}{TN+FP}$$

(11) $$F1=\frac{2TP}{2TP+FP+FN}$$

where TP, TN, FP, and FN denote the number of True Positives, True Negatives, False Positives, and False Negatives, respectively. AUC is the area under the ROC curve.

## 4. Results and Discussion

### 4.1. Classification Performance

Table 3 presents the classification performance of the proposed detection framework. As shown in the results, among the unimodal signals, the ECG modality demonstrates superior performance across most evaluation metrics. This can likely be attributed to the relatively low noise level of the ECG signals and their strong relevance to the target classification task. Notably, ECG achieves the highest specificity (95.06%), indicating its strong discriminative capability in identifying non-target classes. However, its sensitivity is only 77.78%, suggesting that there remains room for improvement in terms of the detection rate. In contrast, the PCG modality exhibits the weakest performance when used independently. Specifically, the results for PCG in terms of F1 score (51.55%), specificity (55.56%), and accuracy (59.83%) indicate relatively poor stability and classification accuracy.

In terms of multimodal fusion, the ECG + EPCG combination achieved strong overall performance across all evaluation metrics. Notably, this fusion strategy yielded the highest sensitivity (80.56%) and F1 score (82.86%) among all tested combinations, indicating a significant advantage in improving detection capability and overall classification performance. Furthermore, its specificity reached 93.83%, demonstrating a well-balanced performance by maintaining high discriminative power for non-target classes. The ECG + EPCG combination also slightly outperformed other combinations in terms of accuracy (89.74%) and AUC (89.08%), highlighting its potential as an effective multimodal input. In contrast, the ECG + PCG combination underperformed, likely due to the relatively poor classification performance of the PCG modality when used alone, which negatively impacted the overall model performance.

Table 4 presents the final fusion classification performance of the model under different convolutional depths. The results indicate that the SCNN model exhibits significant variation in performance depending on the number of convolutional layers. Among them, the 4-layer convolutional architecture used in this study achieves the best overall results, with sensitivity, F1 score, accuracy, and AUC reaching 80.56%, 82.86%, 89.74%, and 89.08%, respectively. This suggests a strong overall classification capability, likely due to a well-balanced trade-off between feature extraction capacity and model complexity at this depth. In contrast, the 3-layer architecture suffers from insufficient depth, resulting in limited feature extraction capability and a relatively low sensitivity of 69.44%, indicating weaker ability to identify target classes. On the other hand, while the 5-layer model achieves the highest specificity (98.77%), its excessive depth may lead to overfitting as reflected by a drop in sensitivity to 63.89%, with overall F1 score and AUC also falling below those of the 4-layer configuration.

Table 5 presents the final fusion classification performance of the model using grayscale and RGB image inputs. The results clearly show that grayscale images significantly outperform colored images across all evaluation metrics. In particular, notable improvements are observed in sensitivity (80.56% vs. 69.44%), F1 score (82.86% vs. 71.43%), accuracy (89.74% vs. 82.91%), and AUC (89.08% vs. 84.12%). This performance gap may be attributed to the fact that grayscale images retain the structural characteristics of the original signals while reducing redundancy and interference introduced by additional color channels. This allows the model to focus more effectively on learning temporal and morphological features. In contrast, while multi-colored images provide additional channel information, they may introduce unnecessary feature complexity, hindering effective feature extraction in convolutional layers and ultimately leading to decreased classification performance.

### 4.2. Analysis of Spike Firing Rate

In ANN, neurons are activated in a continuous manner, and all neurons in each layer participate in every forward pass. Each computation typically requires one floating-point multiplication and one floating-point addition, commonly referred to as a multiply–accumulate operation (MAC). In contrast, SNNs transmit information via discrete binary spikes. A neuron in an SNN only “fires”—i.e., becomes active—when its membrane potential reaches a predefined threshold; otherwise, it remains in a quiescent state. Each such event usually involves only a single floating-point addition. As a result, SNN exhibit sparse activation patterns, offering higher energy efficiency and greater biological plausibility compared to their ANN counterparts. In an SNN architecture, the number of operations can be calculated as follows [28]:

(12) $$OP_{SNN}=Spike\;Rate_{l}\times OP_{ANN}$$

(13) $$\mathrm{Spike}\;{\mathrm{Rate}}_{l}=\frac{\mathrm{Total}\;{\mathrm{Spikes}}_{l}\mathrm{over}\;\mathrm{all}\;\mathrm{inference}\;\mathrm{time}\;\mathrm{steps}}{{\mathrm{Neurons}}_{l}}$$

where $\mathrm{Spike}{\mathrm{Rate}}_{l}$ refers to the total number of spikes generated in layer l across all time steps, divided by the number of neurons in that layer. The overall spike rate is defined as the total number of spikes across all layers and time steps, normalized by the total number of neurons in the entire network. $OP_{ANN}$ denotes the number of operations in an ANN architecture that shares the same structure as the corresponding SNN.

**Figure 3:**
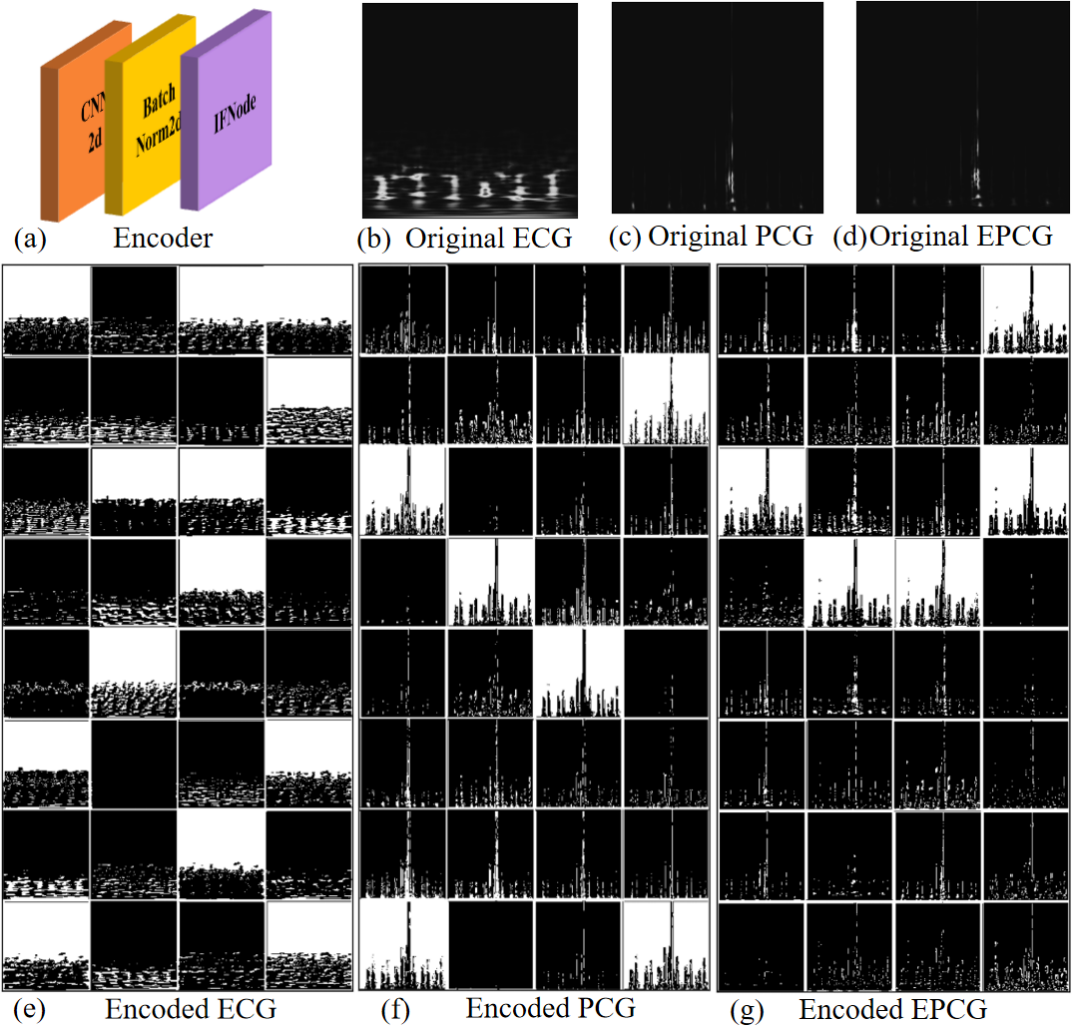
Schematic diagram of model encoding. (**a**) The encoder module of the model; (**b**–**d**) the original ECG, PCG, and EPCG input images, respectively; (**e**–**g**) the encoded output signals of the last time step, respectively (32 channels).

The spike rate plays a critical role in determining the energy efficiency of SNNs compared to ANNs, with lower spike rates corresponding to reduced energy consumption. In this study, we adopt an end-to-end encoding approach to generate binary spike inputs, where input images are directly fed into the model for encoding. Details of the encoding process are illustrated in Figure 3. Specifically, Figure 3a shows the encoder module of the model, which consists of the first three convolutional layers and utilizes IFNode neurons to perform spike-based image encoding. Figure 3b–d and Figure 3e–g show the image data of ECG, PCG, and EPCG signals before and after encoding, respectively. In the post-encoding images, white pixels represent emitted spikes, indicating where neurons have fired during the encoding process.

**Figure 4:**
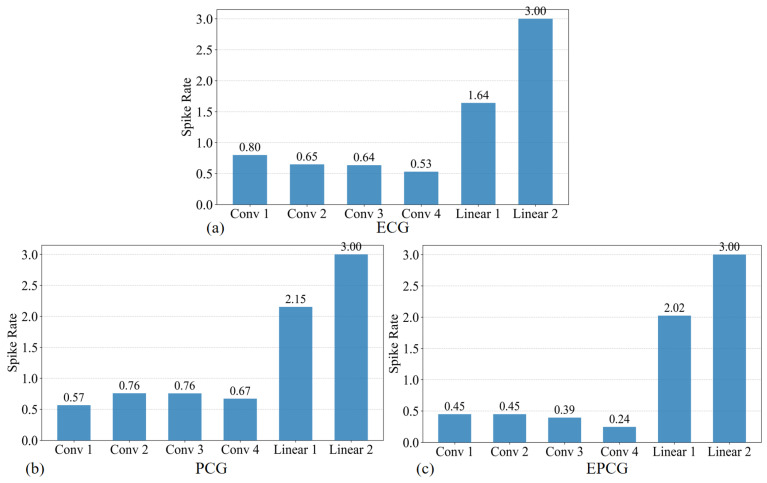
The spike rate of each layer in (**a**) ECG; (**b**) PCG; (**c**) EPCG.

**Figure 5:**
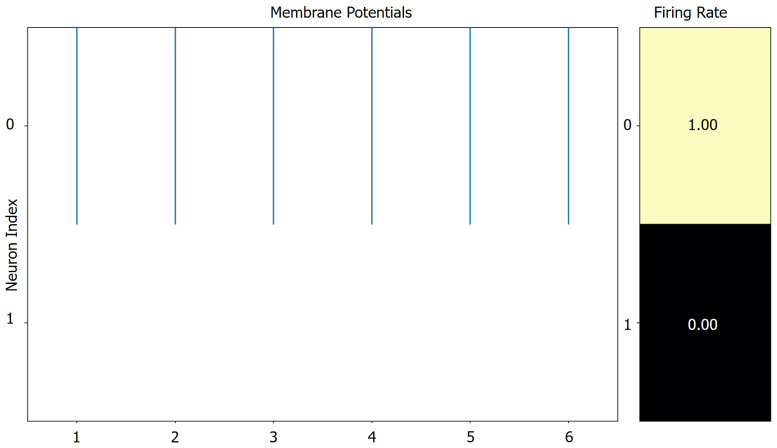
Schematic of the spiking classification output of the model.

**Figure 6:**
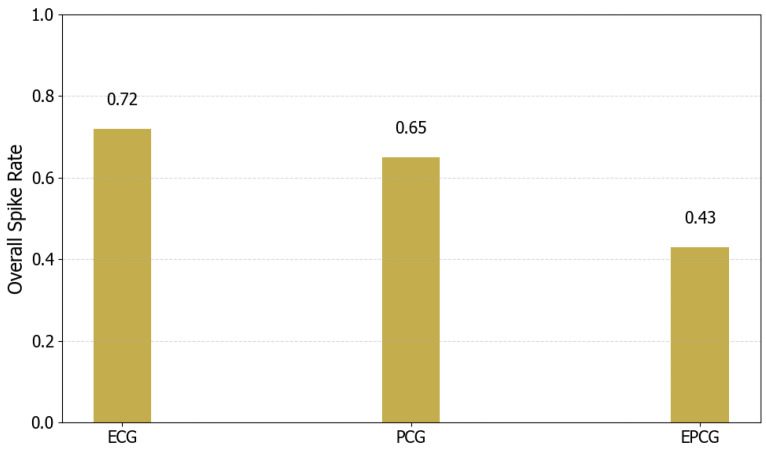
The overall spike rate of ECG, PCG and EPCG.

Next, we computed the spike rate of the model by feeding a single sample, as shown in Figure 4 and Figure 6. Figure 4a–c illustrate the layer-wise spike rates of the model when processing ECG, PCG, and EPCG signals, respectively. It is evident that the EPCG signal consistently produces the lowest spike rate across all convolutional layers, significantly reducing the number of operations. Additionally, the spike rate of the final fully connected layer is 3.0 because the model performs binary classification at each time step, and by design, one of the two output neurons fires in every step. This results in a spike probability of 0.5 per neuron per time step, and over 6 time steps, the total spike count per output neuron sums to 3.0. The model’s spiking classification output is further illustrated in Figure 5.

Figure 6 presents the overall spike rates of the model when processing ECG, PCG, and EPCG signals, respectively. The overall spike rates are 0.72 for ECG, 0.65 for PCG, and 0.43 for EPCG. Notably, the EPCG input leads to the lowest overall spike rate among the three. This reduction may be attributed to the increased information density achieved through multimodal signal fusion, which suppresses unnecessary spike generation and enables the model to achieve more efficient representation and classification with lower energy consumption. These results further demonstrate the energy efficiency advantage of the differential signal-level fusion strategy proposed in this study for SNN-based applications.

### 4.3. Analysis of Energy Consumption

In this study, we compared the energy consumption characteristics of ANN and SNN under a 45 nm CMOS process. As shown in Table 6, the energy consumption of a 32-bit MAC operation in ANN is 4.6 pJ, which is approximately 5.1 times higher than the 0.9 pJ required for an addition operation in SNN. Although the exact values may vary depending on specific process parameters, it is generally observed across most semiconductor technologies that addition operations consume significantly less energy than multiplication operations [28].

Based on Equations (12) and (13) and the data in Table 6, we derived the comparative results presented in Table 7. Using the normalized energy consumption of the ANN as a reference, we calculated the energy efficiency improvements achieved by the SNN. The results show that the SNN achieves 7.1×, 7.9×, and 11.9× energy savings compared to the isomorphic ANN when processing ECG, PCG, and EPCG signals, respectively. These findings demonstrate the low-power advantage of the SNN architecture. Furthermore, the EPCG modality yields the highest energy efficiency due to its lower spike rate, further highlighting the effectiveness of the differential signal-level fusion strategy adopted in this study.

### 4.4. Comparison with Related Research

To more comprehensively assess the contribution of this study, we conducted a comparative analysis with related works as shown in Table 8. In [16], Li et al. utilized ECG and PCG signals with a conventional ANN and feature-level fusion strategy, achieving a classification accuracy of 87.3%. Based on a reimplementation of their model, we estimated its energy consumption to be approximately 153.6 μJ (this is an approximation with potential inaccuracies). Similarly, in [17], Zhu et al. employed a more complex ANN architecture with the same types of input signals and feature-level fusion, resulting in a higher classification accuracy of 91.6% but at the cost of significantly higher energy consumption, approximately 934.7 μJ.

A comparison between these two studies reveals a clear trade-off: simpler models tend to be more energy-efficient but yield lower accuracy, while more complex models achieve better accuracy at the expense of increased energy consumption. In contrast, our method employs ECG and the fused EPCG signal as input, utilizes an SNN architecture, and adopts a decision-level fusion strategy. As a result, our model achieves a classification accuracy of 89.74% with an energy consumption of 209.6 μJ.

Compared with [16], our method improves accuracy by approximately 2.44%, with only a 56 μJ increase in energy consumption. Compared with [17], our model reduces energy consumption by approximately 725.1 μJ, while achieving only a 1.86% lower accuracy. These results indicate that the proposed method strikes a certain balance between energy efficiency and classification performance.

## 5. Conclusions

In this study, a multimodal CVDs detection framework based on SNN is proposed, which effectively integrates ECG and PCG signals through collaborative modeling at both the signal and decision levels. A differential mechanism is employed to fuse ECG and PCG signals at the signal level, generating a composite EPCG signal. The ASLT method is then applied to extract high-resolution time–frequency representations, enabling the fine-grained characterization of both temporal and spectral features. During model training, two SCNNs are trained separately using ECG and EPCG inputs. To enhance overall classification robustness, a CDD fusion strategy is introduced at the decision level. Experiments conducted on the “training-a” of the PhysioNet/CinC Challenge 2016 dataset demonstrate that the proposed framework achieves superior performance and stability compared to single-modality detection methods. Further analysis reveals that both the convolutional depth of the model and the type of input images (grayscale vs. RGB) influence classification outcomes, thereby validating the rationality and effectiveness of the proposed model design. Moreover, a systematic evaluation of spike firing rate and energy consumption is carried out, showing that the proposed method maintains competitive classification accuracy while significantly reducing energy usage, underscoring its potential for deployment in low-power medical diagnostic applications.

In future work, we aim to further optimize the model architecture and fusion strategy, with a focus on exploring the optimal trade-off between energy efficiency and detection performance. Additionally, since we only use the "training-a" dataset, the generalizability and reliability of our study are somewhat limited. In the future, we will consider using more datasets to further strengthen the robustness and persuasiveness of our research.

## Figures and Tables

**Table 1:** Data profile after expansion.

Type	Recordings	Time Length (s)
Negative	804	6
Positive	750	6

**Table 2:** Comparison of average validation loss and overall spike rate at different time steps in ECG and EPCG signals.

Parameters	T = 4 (ECG)	T = 6 (ECG)	T = 8 (ECG)	T = 4 (EPCG)	T = 6 (EPCG)	T = 8 (EPCG)
AVL	0.09	0.08	0.07	0.16	0.14	0.14
OSR	0.40	0.73	0.97	0.25	0.43	0.70

AVL: Average Validation Loss; OSR: Overall Spike Rate.

**Table 3:** Comparison of classification performance between models using multi-modal and single-modal data.

Signal	Sen (%)	F1 (%)	Spe (%)	Acc (%)	Auc (%)
ECG	77.78	82.35	95.06	89.74	89.03
PCG	69.44	51.55	55.56	59.83	65.21
EPCG	50.00	50.70	79.01	70.09	70.66
ECG + PCG	77.78	80.00	92.59	88.03	88.94
ECG + EPCG	80.56	82.86	93.83	89.74	89.08

**Table 4:** Comparison of classification performance of models at different convolution depths based on multimodal data (ECG + EPCG).

Number of Convolution Layers	Sen (%)	F1 (%)	Spe (%)	Acc (%)	Auc (%)
3	69.44	73.53	91.36	84.62	84.33
5	63.89	76.67	98.77	88.03	88.73
4 (Ours)	80.56	82.86	93.83	89.74	89.08

**Table 5:** Comparison of classification performance of the model under different color image inputs based on multimodal data (ECG + EPCG).

Color	Sen (%)	F1 (%)	Spe (%)	Acc (%)	Auc (%)
RGB	69.44	71.43	88.89	82.91	84.12
Grayscale (Ours)	80.56	82.86	93.83	89.74	89.08

**Table 6:** Energy costs of addition and multiplication in 45 nm CMOS [29].

Operation	Energy Consumption
FP ADD (32 bit)	0.9 pJ
FP MULT (32 bit)	0.9 pJ
FP MAC (32 bit)	(0.9 + 3.7) = 4.6 pJ

**Table 7:** Energy consumption improvement of the SNN model in this study over the isomorphic ANN model.

Signal	Normalized ${\mathrm{OP}}_{\mathrm{ANN}}$ (a)	Normalized ${\mathrm{OP}}_{\mathrm{SNN}}$ (b)	ANN/SNN Energy (a×4.6/(b×0.9))
ECG	1.0	0.72	7.1
PCG	1.0	0.65	7.9
EPCG	1.0	0.43	11.9

OP: Number of operation.

**Table 8:** Comparison of the balance between energy consumption and accuracy with related research.

Literature	Signal	OP (M)	Total Energy (μJ)	Acc (%)
[16]	ECG PCG	16.5 16.9	153.6	87.30
[17]	ECG PCG	101.6 101.6	934.7	91.60
Ours	ECG EPCG	145.8 87.1	209.6	89.74

OP: Number of operation.

## Data Availability

Data are contained within the article.

## References

[1] Humayun A.I., Ghaffarzadegan S., Ansari M.I., Feng Z., Hasan T. (2020). Towards domain invariant heart sound abnormality detection using learnable filterbanks. IEEE J. Biomed. Health Inform..

[2] Gregory A.R., George A.M., Catherine O.J., Giovanni A., Enrico A., Larry M.B., Noel C.B., Andrea Z.B., Emelia J.B., Catherine P.B. (2020). Global burden of cardiovascular diseases and risk factors, 1990–2019: Update from the GBD 2019 study. J. Am. Coll. Cardiol..

[3] Dey M., Omar N., Ullah M.A. (2021). Temporal feature-based classification into myocardial infarction and other CVDs merging CNN and Bi-LSTM from ECG signal. IEEE Sens. J..

[4] Baloglu U.B., Talo M., Yildirim O., Tan R.S., Acharya U.R. (2019). Classification of myocardial infarction with multi-lead ECG signals and deep CNN. Pattern Recognit. Lett..

[5] Hannun A.Y., Rajpurkar P., Haghpanahi M., Tison G.H., Bourn C., Turakhia M.P., Ng A.Y. (2019). Cardiologist-level arrhythmia detection and classification in ambulatory electrocardiograms using a deep neural network. Nat. Med..

[6] Yao Q., Wang R., Fan X. (2020). Multi-class arrhythmia detection from 12-lead varied-length ECG using Attention-based Time-Incremental Convolutional Neural Network. Inf. Fusion.

[7] Ghaffari A., Madani N. (2019). Atrial fibrillation identification based on a deep transfer learning approach. Biomed. Phys. Eng. Express.

[8] Clifford G.D., Liu C., Moody B., Millet J., Schmidt S., Li Q., Silva I., Mark R.G. (2017). Recent advances in heart sound analysis. Physiol. Meas..

[9] Tang H., Dai Z., Jiang Y., Li T., Liu C. (2018). PCG classification using multidomain features and SVM classifier. BioMed Res. Int..

[10] Li F., Liu M., Zhao Y., Kong L., Dong L., Liu X., Hui M. (2019). Feature extraction and classification of heart sound using 1D convolutional neural networks. EURASIP J. Adv. Signal Process..

[11] Acharya U.R., Fujita H., Oh S.L., Hagiwara Y., Tan J.H., Adam M. (2017). Automated detection of arrhythmias using different intervals of tachycardia ECG segments with convolutional neural network. Inf. Sci..

[12] Zhu W., Chen X., Wang Y., Wang L. (2018). Arrhythmia recognition and classification using ECG morphology and segment feature analysis. IEEE/ACM Trans. Comput. Biol. Bioinform..

[13] Kui H., Pan J., Zong R., Yang H., Wang W. (2021). Heart sound classification based on log mel-frequency spectral coefficients features and convolutional neural networks. Biomed. Signal Proces..

[14] Kusuma S., Jothi K.R. (2022). ECG signals-based automated diagnosis of congestive heart failure using deep CNN and LSTM architecture. Biocybern. Biomed. Eng..

[15] Alkhodari M., Fraiwan L. (2021). Convolutional and recurrent neural networks for the detection of valvular heart diseases in phonocardiogram recordings. Comput. Methods Prog. Biomed..

[16] Li P., Huang Y., Liu Z.P. (2021). Prediction of cardiovascular diseases by integrating multi-modal features with machine learning methods. Biomed. Signal Proces..

[17] Zhu J., Liu H., Liu X., Chen C., Shu M. (2025). Cardiovascular disease detection based on deep learning and multi-modal data fusion. Biomed. Signal Proces..

[18] Rana A., Kim K.K. (2021). A novel spiking neural network for ECG signal classification. J. Sens. Sci. Technol..

[19] Zhu R.J., Zhang M., Zhao Q., Deng H., Duan Y., Deng L.J. (2025). TCJA-SNN: Temporal-channel joint attention for spiking neural networks. IEEE Trans. Neural Netw. Learn. Syst..

[20] Roy K., Jaiswal A., Panda P. (2019). Towards spike-based machine intelligence with neuromorphic computing. Nature.

[21] Stromatias E., Neil D., Pfeiffer M., Galluppi F., Furber S.B., Liu S.C. (2015). Robustness of spiking deep belief networks to noise and reduced bit precision of neuro-inspired hardware platforms. Front. Neurosci..

[22] Rana A., Kim K.K. (2024). Electrocardiography classification with leaky integrate-and-fire neurons in an artificial neural network-inspired spiking neural network framework. Sensors.

[23] Liu C., Springer D., Li Q., Moody B., Juan R.A., Chorro F.J., Castells F., Roig J.M., Silva I., Johnson A.E.W. (2016). An open access database for the evaluation of heart sound algorithms. Physiol. Meas..

[24] Liu C., Springer D., Clifford G.D. (2017). Performance of an open-source heart sound segmentation algorithm on eight independent databases. Physiol. Meas..

[25] Li H., Wang X., Liu C., Wang Y., Li P., Tang H., Yao L., Zhang H. (2019). Dual-input neural network integrating feature extraction and deep learning for coronary artery disease detection using electrocardiogram and phonocardiogram. IEEE Access.

[26] Muresan V.V., Braband H., Neacsu A., Martin R.C. (2021). Time-frequency super-resolution with superlets. Nat. Commun..

[27] Fang W., Chen Y., Ding J., Yu Z., Masquelier T., Chen D., Huang L., Zhou H., Li G., Tian Y. (2023). SpikingJelly: An open-source machine learning infrastructure platform for spike-based intelligence. Sci. Adv..

[28] Rathi N., Roy K. (2023). DIET-SNN: A low-latency spiking neural network with direct input encoding and leakage and threshold optimization. IEEE Trans. Neural Netw. Learn. Syst..

[29] Horowitz M. (2014). 1.1 Computing’s energy problem (and what we can do about it). Proceedings of the IEEE International Solid-State Circuits Conference Digest of Technical Papers (ISSCC).

